# p47phox siRNA-Loaded PLGA Nanoparticles Suppress ROS/Oxidative Stress-Induced Chondrocyte Damage in Osteoarthritis

**DOI:** 10.3390/polym12020443

**Published:** 2020-02-13

**Authors:** Hyo Jung Shin, Hyewon Park, Nara Shin, Hyeok Hee Kwon, Yuhua Yin, Jeong-Ah Hwang, Song I Kim, Sang Ryong Kim, Sooil Kim, Yongbum Joo, Youngmo Kim, Jinhyun Kim, Jaewon Beom, Dong Woon Kim

**Affiliations:** 1 Department of Medical Science, Chungnam National University College of Medicine, Daejeon 35015, Korea; shinhyo1013@gmail.com (H.J.S.); phw6304@gmail.com (H.P.); s0870714@gmail.com (N.S.); kara00124@gmail.com (H.H.K.); yoonokhwa527@gmail.com (Y.Y.); ijjanghwang@gmail.com (J.-A.H.); kthddl2295@gmail.com (S.I.K.); 2 Department of Anatomy and Cell Biology, Brain Research Institute, Chungnam National University College of Medicine, Daejeon 35015, Korea; sikim@cnu.ac.kr; 3 School of Life Sciences, BK21 Plus KNU Creative BioResearch Group, Institute of Life Science & Biotechnology, Brain Science and Engineering Institute, Kyungpook National University, Daegu 41566, Korea; srk75@knu.ac.kr; 4 Department of Orthopedics, Chungnam National University College of Medicine, Daejeon 35015, Korea; longman76@hanmail.net (Y.J.); osdr69@cnuh.co.kr (Y.K.); 5 Division of Rheumatology, Department of Internal Medicine, Chungnam National University College of Medicine, Daejeon 35015, Korea; md228@hanmail.net; 6 Department of Physical Medicine and Rehabilitation, Chung-Ang University Hospital, Chung-Ang University College of Medicine, Seoul 06973, Korea; powe5@cau.ac.kr

**Keywords:** osteoarthritis, monosodium iodoacetate, p47phox, PLGA nanoparticles, reactive oxygen species

## Abstract

Osteoarthritis (OA) is the most common joint disorder that has had an increasing prevalence due to the aging of the population. Recent studies have concluded that OA progression is related to oxidative stress and reactive oxygen species (ROS). ROS are produced at low levels in articular chondrocytes, mainly by the nicotinamide adenine dinucleotide phosphate (NADPH) oxidase, and ROS production and oxidative stress have been found to be elevated in patients with OA. The cartilage of OA-affected rat exhibits a significant induction of p47phox, a cytosolic subunit of the NADPH oxidase, similarly to human osteoarthritis cartilage. Therefore, this study tested whether siRNA p47phox that is introduced with poly (D,L-lactic-co-glycolic acid) (PLGA) nanoparticles (p47phox si_NPs) can alleviate chondrocyte cell death by reducing ROS production. Here, we confirm that p47phox si_NPs significantly attenuated oxidative stress and decreased cartilage damage in mono-iodoacetate (MIA)-induced OA. In conclusion, these data suggest that p47phox si_NPs may be of therapeutic value in the treatment of osteoarthritis.

## 1. Introduction

Osteoarthritis (OA) is the most prevalent form of joint disease, a hallmark of which is cartilage loss [1]. However, OA also affects various tissues and ultimately triggers articular chondrocyte degeneration, chondrocyte clustering, synovial inflammation, osteophyte formation, and subchondral bone remodeling in affected articular cartilage [2,3,4]. Patients with degenerative arthritis exhibit varying degrees of oxidative stress and cartilage destruction [5]. Additionally, reactive oxygen species (ROS) (in particular chondrocytes) are produced at low levels, mainly by the nicotinamide adenine dinucleotide phosphate (NADPH) oxidase, where they act as integral mediators of intracellular signaling mechanisms that contribute to the maintenance of cartilage homeostasis [6]. ROS production and oxidative stress are elevated in patients with OA; compared to normal cartilage, OA cartilage has a significantly greater degree of ROS-induced DNA damage, which is mediated by interleukin-1 [7]. In contrast, the levels of antioxidant enzymes (e.g., superoxide dismutase, catalase, and glutathione peroxidase) are lower in patients with OA, which indicates that oxidative stress plays a role in OA pathogenesis [8,9].

The NOX family of NADPH oxidases is a major source of ROS production under various pathological conditions. Currently, this family is known to include seven members that utilize various combinations of subunits to form active enzyme complexes: NOX1, NOX2, NOX3, NOX4, NOX5, dual oxidase 1 (DUOX1), and DUOX2 [10,11,12]. Of these combinations, the NOX2 complex is composed of two membrane-bound subunits (p22phox and gp91phox), various cytosolic proteins (p40phox, p47phox, and p67phox), and an Rac1 GTPase; these components assemble at membrane sites upon cell activation. Recently, phagocyte-type NADPH oxidases have been identified in other cell types (e.g., adventitial fibroblasts, vascular smooth muscle cells, endothelial cells, and renal mesangial cells), where this enzyme is thought to serve a signaling function [13].

Upon exposure to cytokines, synovial NOX is known to produce superoxide anions that activate multiple signaling pathways and lead to the expression of inflammatory genes in rheumatoid arthritis synovial fibroblasts [14,15,16]. Many studies have suggested that excessive levels of NOX-mediated ROS generation appear to be a major mediator of the pathogenesis of OA. For example, NOX4/p22phox-induced ROS production plays an essential role in the chondrocytes of patients with OA [17,18,19,20]. Moreover, a recent study showed that, compared to a control group, patients with knee OA exhibited 4.8- and 8.4-fold increases in the protein contents of prolidase and NOX2, respectively, while xanthine oxidase levels tended to increase, and a 5.4-fold increase was observed in NALP3 inflammasomes [21]. The van Dan Bosch group reported that NOX2-derived ROS enhance joint destruction during collagenase-induced OA [22], while another study showed that NOX2 is involved in the production of superoxides in synovial cells that were obtained from patients with rheumatoid arthritis and patients with OA [23]. Furthermore, NOX2-generated ROS are involved in chondrocyte death that is induced by interleukin-1β and the loss of the extracellular matrix due to the activation of hyaluronidase via acidification [24,25,26]. However, to the best of our knowledge, the role of p47phox, an NADPH oxidase subunit that is the major NOX that is involved in OA pathogenesis, has not yet been fully elucidated. It is known that p47phox plays a pivotal role in neutrophil NOX2 activation by providing domains for physical binding to cytochrome b558 (a complex that is associated with gp91phox and p22phox) and p67phox [27,28].

Local treatment via intra-articular injection is an appropriate strategy due to the fact that OA only affects the joints. When small molecular drugs have been introduced into intra-articular space, they have been easily and quickly removed by blood vessels and lymphatics [29]. Therefore, a suitable drug delivery system, such as biodegradable and bioeliminable material nanoparticles, is required for these drugs to increase their solubility and prolong their retention time in the articular cavity. In this study, we adopted poly (D,L-lactic-co-glycolic acid) (PLGA) polymers as a carrier system, because they have shown the ability to protect the loaded drug or siRNA from inactivation, reduce unwanted side effects, and enhance the efficacy of the active pharmaceutical ingredient due to improved solubility and bioavailability. Notably, only nanoparticles (NPs) with a size of less than 200 nm have the ability to easily permeate through mucus without being immobilized by the natural size-filtering mechanism [30].

Thus, the present study investigated the roles of p47phox in ROS production and cartilage damage in an OA model by using NPs.

## 2. Materials and Methods

### 2.1. Animals and Osteoarthritis Model

Male Sprague–Dawley rats weighing 120–140 g at the time of OA induction were used. Under brief isoflurane anesthesia, the animals were intra-articularly injected in the left knee with 2 mg of mono-iodoacetate (MIA; #I2512; Sigma-Aldrich, St. Louis, MI, USA) that was dissolved in 20 µL saline; controls received saline only (day 0). MIA was delivered through the left patellar tendon by using a 30-G needle.

### 2.2. Human Chondrocyte

We used a scalpel to excise cartilage from the femoral condyles and posterior patellar surfaces of OA patients that were treated at Chungnam National University Hospital (CNUH) (approval no. IRB-2016-06-007). The cartilage was cut into 2 mm thick pieces, and the digested suspension was passed through a 40 µM pore-size cell strainer to isolate individual chondrocytes. Cells were counted by using a cell counter. Cells (5 × 10^6^) were seeded into 10 mm diameter dishes and cultured for 10 days in Dulbecco’s minimal essential medium (DMEM) supplemented with 10% (*v*/*v*) fetal bovine serum (FBS). The medium was changed every 2 days [31].

### 2.3. Hematoxylin and Eosin Staining

Cartilage from humans with OA was frozen, sectioned to a 4-μm thickness, and fixed in 4% paraformaldehyde (PFA). MIA-injected knees were fixed in 4% (*v*/*v*) paraformaldehyde for 2 days, decalcified in a Calci-Clear solution (catalog no. HS-105; National Diagnostics, Atlanta, GA, USA) for 2 days, sectioned in the coronal plane (4 µm thickness), embedded in paraffin wax, and used to prepare slides after staining with hematoxylin. Then, the slides were sequentially dehydrated in 70%, 80%, 90%, and 100% ethanol. Finally, sections were cleared in xylene. A light microscope and a digital camera were used to capture and evaluate the histopathological features of the articular cartilage.

### 2.4. Behavior Test

Mechanical paw withdrawal thresholds were measured via up–down von Frey testing [32]. Rats were placed on an elevated metal grid. Fifty percent withdraw threshold values were determined by using the up–down method. Briefly, mechanical allodynia was assessed by measuring foot withdrawal thresholds in response to mechanical stimuli to the hind paw. The withdrawal threshold was determined by using the up–down method with a set of von Frey filaments from 0.008 to 1.4 g (0.008, 0.02, 0.04, 0.07, 0.16, 0.4, 0.6, 1, and 1.4 g).

### 2.5. Immunohistochemistry

After incubating the tissues with a blocking buffer (5% normal serum/0.3% Triton X-100, Bio-Rad, Irvine, CA, USA) for 1 h to prevent nonspecific binding, the sections were incubated with primary antibodies (p47phox, #sc-17844, 1:200, SantaCruz, Dallas, TX, USA) and diluted in a blocking buffer. Immunostaining was performed by using the avidin–biotin peroxidase complex (ABC) method, as reported previously [33,34]. The sections were mounted with Vectashield (Vector Laboratories, Burlingame, CA, USA), and images were obtained with a confocal microscope. The immunodensities in the graphs were quantified by the Image J program software.

### 2.6. DHE Staining

Superoxide anion levels in the spinal cord were determined by using dihydroethidium (DHE; Thermo Fisher Scientific), as described previously [34]. Cartilage sections were incubated with DHE (1 μM) at room temperature for 5 min and mounted on slides. 

### 2.7. Cytotoxicity Assay

An MTT assay was performed in order to test the cell viability caused by siRNA-encapsulated PLGA nanoparticles as per the manufacturer’s instructions. Detailed procedures can also be found in previous reports [35].

### 2.8. Preparation of siRNA-Encapsulated PLGA Nanoparticles

p47phox siRNA-encapsulated PLGA NPs were prepared from a PLGA nanoparticle synthesis service from the Nanoglia company (Daejeon, Republic of Korea) with minor modifications, as reported previously [33,35]. A copolymer of DL-lactic and glycolide in a 50/50 molar ratio and with an inherent viscosity midpoint of 0.2 dL/g was used. To produce p47phox siRNA-encapsulated PLGA NPs, 200 μL of 200 μM siRNA of a TE 8.0 buffer was added in drops to 800 μL of dichloromethane (DCM) that contained 25 mg of PLGA (Corbion, Amsterdam, the Netherlands) and then emulsified by sonication (50 W, 1 min; Vibra-Cell™ VCX 130; Sonics, Newtown, CT, USA) into a primary W1/O emulsion. Later, 2 mL of 1% PVA1500 (*w*/*v*; Thermo Fisher Scientific, Waltham, MA, USA) was directly added into the primary emulsion and further emulsified by sonication for 1 min to form a W1/O/W2 double emulsion. The resulting product was then diluted with 6 mL of 1% PVA1500 (*w*/*v*) and stirred magnetically for 3 h at room temperature to evaporate the DCM in a fume hood. Finally, the PLGA NPs were collected by centrifugation at 15,000× *g* for 15 min at 4 °C, washed twice with deionized water, and freeze-dried with 10 vials.

### 2.9. Colloidal Characterization of Nanoparticles

Two milligrams of lyophilized particles were dispersed in 1 mL of deionized water to determine the size distribution, zeta potential and polydispersity index (PDI) with the Zetasizer Nano ZS (Malvern Instruments, Malvern, UK), and the diameter and shape were determined with scanning electron microscopy (SEM; SNE-4500 M; SEC Co., Ltd., Suwon, Korea). The each 20 µM of siRNA-encapsulated PLGA nanoparticles, were collected and incubated in a Eppendorf tube with 250 µL of PBS, incubated at 37 °C for 48 h. At the designated time, 200 µL of the released medium was taken and replaced by the same amount of a fresh buffer. The amount of the p47phox siRNA was measured in the released buffer by using NanoDrop (Thermo Fisher Scientific). The accumulated release percentage of the p47phox siRNA and the entrapment efficiency were evaluated according to a previous report [36].

### 2.10. Statistical Analysis

The data are expressed as mean ± standard error of mean (SEM). The statistical significance between multiple groups was compared by a one-way analysis of variance (ANOVA) followed by an appropriate multiple comparison test. *p*-values of less than 0.05 were considered statistically significant. All statistical analyses were performed by using GraphPad Prism 6 (GraphPad Software Inc., San Diego, CA, USA).

## 3. Results

### 3.1. p47phox and ROS Were Highly Expressed in Chondrocyte Clusters in Human OA Tissues

**Figure 1:**
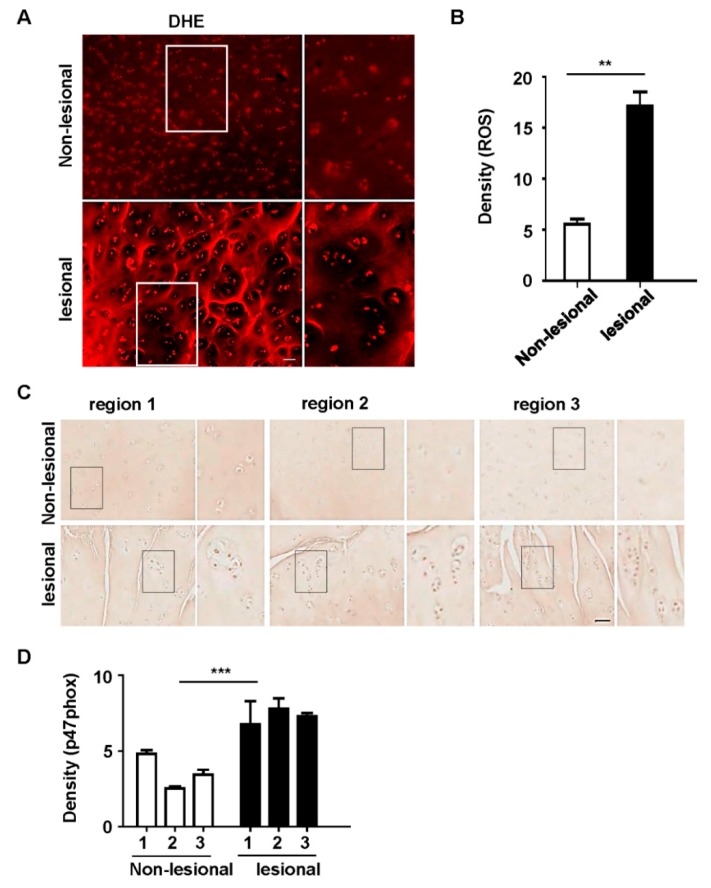
Reactive oxygen species (ROS) production and p47phox expression increased in the articular cartilage of human osteoarthritis (OA) knee joints. (**A**) Representative images of ROS-dependent dihydroethidium (DHE) fluorescence in human OA knee joints. (**B**) DHE staining density measured by using ImageJ software. (**C**) p47phox exhibited prominent expression at lesional sites, compared to non-lesional sites; immunohistochemical analyses show the expression levels of p47phox in knee cartilage at different sites. (**D**) p47phox immunostaining density quantified by ImageJ software; scale bar = 50 µm.

Chondrocyte clustering is a histological sign of late-stage OA [1,37]; this type of clustering and its associated morphological abnormalities are particularly evident in the superficial zones of OA tissues. To explore whether p47phox was associated with ROS production and cartilage damage, samples of degenerating articular cartilage from patients with OA were assessed. In osteoarthritic joint cartilage, cluster cell numbers exceeded eight in affected lacunae, but this did not occur in non-affected lacunae (Figure 1A,B); DHE staining revealed an increased ROS production at injured sites, relative to the less involved areas (Figure 1A,B). Immunohistochemistry analyses of p47phox also revealed high levels of p47phox expression in chondrocytes in joint cartilage samples with clusters (Figure 1C,D). Taken together, these results indicated that increased levels of ROS production and p47phox were associated with cartilage degeneration in patients with OA.

### 3.2. p47phox Was Highly Expressed in the Chondrocytes of MIA-Induced OA Rats

The progression of OA is significantly related to oxidative stress and ROS [7]. The major sites of ROS generation include the mitochondria (via oxidative phosphorylation), the non-mitochondrial membrane-bound NADPH oxidase, and the xanthine oxidase [38]. To verify the effects of the NADPH oxidase on OA progression, the present study examined the expression levels of five components of the NADPH oxidase: three cytosolic fractions (p40phox, p47phox, and p67phox) and two membrane fractions (p22phox and gp91phox). As proof-of-concept, monosodium iodoacetate (MIA) was injected into the knees of rats to assess changes in the levels of NADPH oxidase components in an animal model of toxin-induced OA.

**Figure 2:**
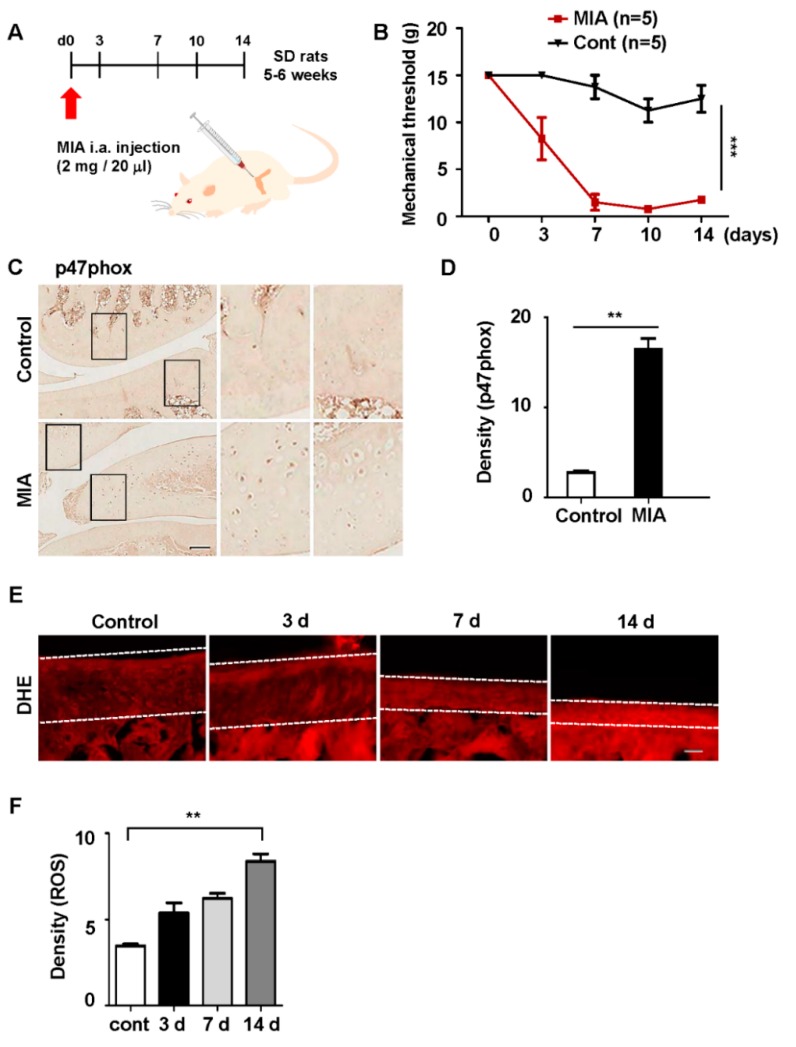
Monosodium iodoacetate (MIA)-induced expression of p47phox and the production of cellular ROS. (**A**) Prior to MIA injection, the rats were subjected to a von Frey filament test; only those that met a predefined threshold were selected for MIA injections. (**B**) The von Frey test was repeated on days 3, 7, 10, and 14 after injection; all data are presented as the mean ± standard error of the mean. (**C**) Rat knee tissues were immunostained with an anti-p47phox antibody at 3 days after MIA injection; scale bar = 50 µm. (**D**) The density of p47phox expression in the knee was measured by using ImageJ software; all data are presented as the mean ± standard error of the mean. (**E**) DHE fluorescence imaging of the knee in the OA rat model; white lines indicate cartilage in the tissue. (**F**) Quantification of DHE fluorescence; all data represent the mean ± standard error of the mean (error bars) of three experiments.

The most common experimental OA model features intra-articular injections of MIA, which is a metabolic inhibitor, into the knee joints of rats [39]. This process results in the progressive loss of articular cartilage and the development of subchondral bone lesions; these closely resemble clinical findings in patients with OA. In the present study, a preliminary experiment was conducted by using a 2 mg/20 µL dose of MIA, which revealed that articular cartilage loss from the extracellular matrix was more pronounced on day 3 (data not shown). These results showed that MIA induced significant cartilage damage in the medial tibial plateau and femoral condyle at day 3 after intra-articular injection. Additionally, mechanical hypersensitivity was observed after approximately seven days and persisted at 14 days in the MIA-treated group (Figure 2B).

Because the cartilage damage that was caused by MIA was associated with ROS production, the expression levels of p47phox were measured. Animals injected with 2 mg of MIA exhibited an increased expression of p47phox by day 3 (Figure 2C,D). Next, the effects of MIA on ROS levels in chondrocytes were investigated; ROS production significantly increased in the cartilage of the MIA-treated group (Figure 2E,F). Taken together, these data suggested that the upregulation of p47phox in cartilage was associated with articular cartilage loss and ROS production. 

### 3.3. Colloidal Characterization of p47phox siRNA-Encapsulated PLGA NPs 

The present study also assessed whether the inhibition of p47phox would affect cartilage damage in the rat model of MIA-induced OA. The efficient delivery of p47phox siRNA into the joints by using a gene delivery system requires the consideration of several factors. Thus, the present study employed poly(D,L-lactic-co-glycolic acid) (PLGA) copolymers; these are biodegradable in and biocompatible with humans, and some products from PLGA are widely used in many Food and Drug Administration (FDA)-approved drugs. There are currently 15 FDA-approved PLA/PLGA-based drug products that are available on the US market [40].

**Figure 3:**
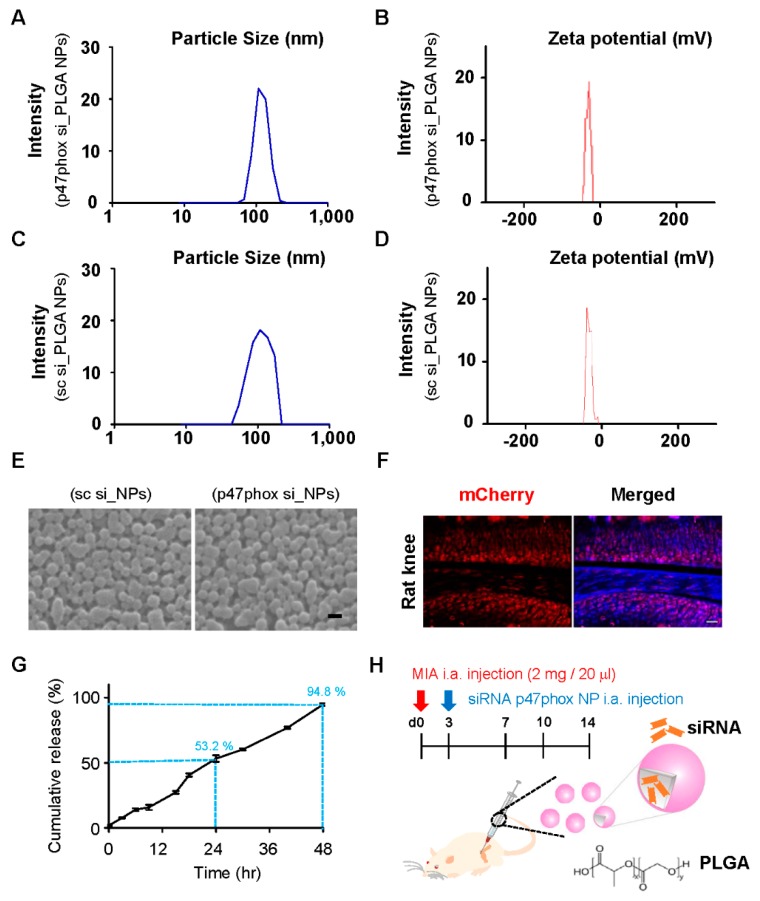
Characterization of p47phox siRNA-loaded poly (D,L-lactic-co-glycolic acid) (PLGA) nanoparticles (NPs). siRNA p47phox-encapsulated PLGA nanoparticles were dissolved in water and measured in terms of (**A**,**C**) size and (**B**,**D**) zeta potential by using a Zetasizer ZS90. (**E**) Suspended NPs were also assessed by using scanning electron microscopy; scale bar = 200 nm. (**F**) After 3 days, normal rat knees received intra-articular injections of AAV-mCherry expression vector-loaded PLGA NPs, and they were then were examined under a fluorescent microscope to assess uptake; scale bar = 50 µm. (**G**) In vitro cumulative siRNA release of PLGA NPs over 48 h. (**H**) Experimental schematic of the present study that used an MIA-induced OA animal model.

Our research group previously demonstrated that p38 MAPK siRNA that is encapsulated in PLGA NPs attenuates spinal nerve ligation-induced neuropathic pain [35]. Moreover, our group reported that Foxp3 plasmid-loaded PLGA NPs can effectively relieve neuropathic pain in animals by reducing microglia activity and subsequently modulating neuroinflammation [33]. For the present study, p47phox siRNA-loaded PLGA NPs (p47phox si_PLGA NPs) were prepared via sonication by using the double emulsion (W/O/W) method. To prepare PLGA nanoparticles, 200 µL of 20 µM siRNA or scrambled siRNA in a TE7.5 buffer was added to 800 µL of dichloromethane (DCM) that contained 25 mg of PLGA. The average size and zeta potential of siRNA p47phox NPs were 126 ± 55 nm and −23 ± 2 mV, respectively (Figure 3A,B), and those of scrambled siRNA-encapsulated NPs were 117 ± 52 nm and −20 ± 2 mV, respectively, when measured with a Zetasizer ZS90 (Figure 3C,D). In addition, Zetasizer measurements revealed the formation of monodisperse particles (PDI ≤ 0.2) within the desired size range on scrambled or siRNA encapsulation. It should be noted that although PDI values smaller than 0.3 are considered acceptable for drug delivery applications, more specific standards and guidelines have yet to be established by regulatory authorities [30,41]. Furthermore, the uniformity and morphology of the NPs were confirmed by using scanning electron microscopy (Figure 3E). Prior to the in vivo administration of p47phox NPs to the MIA-induced OA rats, the present study assessed whether PLGA NPs would preferentially localize to articular cartilage. To accomplish this, PLGA NPs that encapsulated plasmid-expressing mCherry (pAAV-EF1a-MCS-T2A-mCherry) were administered into the knee joints of rats [33]; on post-injection day 3, mCherry was observed in the cartilage (Figure 3F). 

Next, the p47phox siRNA release profiles from p47phox si_NPs were analyzed by using a cumulative percentage approach. The results showed that p47phox si_NPs gradually released siRNA; this cumulative release peaked at 48 h after injection. After an initial burst of release (53.2%) at 24 h, the release of siRNA was consequently observed in a sustained manner at different times. The percentage of encapsulation efficiency was calculated as the amount of siRNA released from the lyophilized PLGA NPs/the amount of siRNA initially taken to prepare the NPs × 100. Encapsulation efficiency was 36.4 ± 0.17% (Figure 3G). We set up the in vivo administration of p47phox si_NPs in the OA model via intra-articular injections at three days after MIA treatment (Figure 3H).

### 3.4. Inhibition of p47phox by NP-Delivered siRNA Attenuated Pain Behaviors, Cartilage Damage, and ROS Production in Knee Joints with MIA-Induced OA

**Figure 4:**
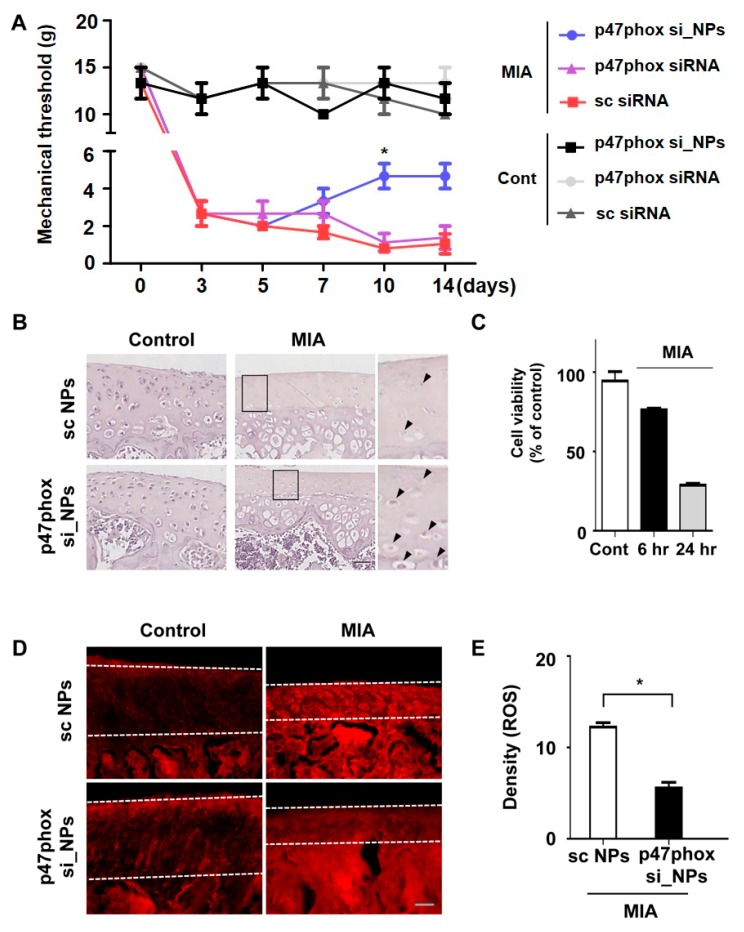
p47phox siRNA-encapsulated PLGA NPs reduced chondrocyte cell death by decreasing ROS production in OA rats. (**A**) On day 3 after MIA-induced OA, p47phox siRNA-loaded NPs were directly administered to the knee via intra-articular injections, and von Frey filament tests were performed on days 7, 10, and 14 after injection; all data are presented as the mean ± standard error of the mean. (**B**) Representative hematoxylin-stained sections of knee joints from rats with MIA-induced OA after 14 days, scale bar = 50 μm. (**C**) Cell viability over time in human primary chondrocytes following MIA injection. (**D**) DHE fluorescence imaging of knees in the OA rat model at 1 week after injection of PLGA NPs; white lines indicate cartilage in the tissue, scale bar = 50 μm. (**E**) Density of fluorescence intensity showing ROS production; all data represent the mean ± standard error of the mean (error bars) of three experiments.

To address the question whether siRNA-encapsulated nanoparticles could work in cartilage, the determination of the effective dose was firstly performed. Different doses of siRNA p47phox NPs—0.2, 0.4, and 0.8 µM—were applied to the MIA-induced OA (Supplementary Figure S1A). The mechanical thresholds were reduced in all dose dependent groups. As the lowest dose of 0.2 µM could reduce pain hypersensitivity, we used it for next investigations. Next, PLGA NPs that contained scrambled siRNA or p47phox siRNA were delivered to the cartilage via intra-articular injections. Because subchondral changes are closely associated with pain and are predictive of the severity of cartilage damage in OA [42], the present study assessed whether p47phox si_NPs could attenuate OA-related pain behavior by using the von Frey filament test. Compared to saline controls, MIA injections (2 mg/20 µL) induced mechanical allodynia in the ipsilateral paw. However, injections of p47phox si_PLGA NPs alleviated mechanical allodynia in MIA rats for up to 14 days after injection (Figure 4A). 

Next, the present study investigated whether p47phox si_NPs could attenuate the loss of proteoglycan and calcification of articular cartilage on day 3 after MIA injection. Compared to scrambled siRNA-loaded NPs, treatment with p47phox si_NPs reduced the thickness of the subchondral bone plate and attenuated the loss of cartilage lacunae (Figure 4B). To observe whether MIA treatment affected cell viability, the human primary chondrocyte cells were incubated alone or in the presence of 5 µM MIA for 6 or 24 h. Following treatment, the viability of the cells was decreased to approximately 30% of their pre-treatment levels (Figure 4C). These results further support the idea that MIA-mediated OA could increase chondrocytic cell death in OA, and ROS production was examined in the MIA-injected joints of rats that received p47phox si_NPs. DHE staining revealed that administration of p47phox si_NPs attenuated ROS production in cartilage (Figure 4D,E). Taken together, these findings suggested that the inhibition of p47phox attenuated pain behaviors, cartilage damage, and ROS production in knee joints with MIA-induced OA.

## 4. Discussion

The signaling pathways by which ROS contribute to the pathophysiology of OA are complex and require further investigation. In general, antioxidant therapies are inefficient treatments for the relief of OA symptoms, whereas antioxidant drugs have shown promising in vitro results; thus, further human studies are required. 

In the present study, MIA-induced OA chondrocytes produced ROS, a result that is consistent with findings from MIA-treated human chondrocytes. Excessive ROS production causes apoptotic cell death in OA chondrocytes; once induced, ROS are synthesized at a constant rate for a substantial period of time. The present results demonstrated a marked increase in the release of ROS in MIA-induced OA chondrocytes. Excessive levels of intracellular ROS are due to oxidative stress and enhanced levels of oxidative markers, which are the primary causes of cell damage and death. Thus, the inhibition of ROS-mediated injuries can reduce oxidative stress, protect chondrocytes, and treat OA. The present results demonstrated that the suppression of MIA-mediated injuries attenuated oxidative stress and protected chondrocytes. Therefore, the present findings contribute to the expanding understanding of proteins that are involved in the regulation of p47phox activation, and they also provide further insights regarding possible mechanisms that are involved in the regulation of cellular fates following OA.

The permeability transition pore is generally regarded as the major ROS target inside mitochondria [43]. The oxidative modification of mitochondrial permeability transition pore proteins has a significant impact on mitochondrial anion fluxes. For example, in response to pro-apoptotic stimuli, including ROS overload, mitochondrial permeability transition pores assume a high-conductance state that allows for the deregulated entry of small solutes into the mitochondrial matrix along their electrochemical gradients. The redox regulation of proteins by moderate levels of ROS has been observed in various signaling pathways, including the autophagy pathway, which is a catabolic pathway for the degradation of intracellular proteins and organelles via lysosomes. Some studies have shown that autophagy and cartilage damage are increased in MIA-induced OA models [44], while others have shown that OA chondrocytes exhibit reduced mitochondrial membrane potentials. Thus, the present study aimed to further clarify that these findings were not due to the blockage of autophagy.

The activation and regulation of the NADPH oxidase are controlled by the phosphorylation of its cytosolic component (p47phox) on serine subunits that are located between Ser303 and Ser379 [45]. Notably, the stimulation of neutrophils by high concentrations of the chemotactic peptide n-formyl methionyl-leucyl-phenylalanine, due to the protein kinase C agonist (phorbol 12-myristate 13-acetate), induces the complete phosphorylation of p47phox, which is required for the activation of the NADPH oxidase [46]. The present study showed that p47phox phosphorylation was required for MIA-induced OA. During the activation of the NADPH oxidase, approximately 10%–20% of p47phox proteins migrate to the plasma membrane, whereas 80%–90% remain in the cytosol. During activation, p47phox is presumed to bind to gp91phox/NOX2 and p22phox, because the translocation of p47phox to the plasma membranes impairs neutrophils from gp91phox- or p22phox-deficient patients. Though gp91phox is the central docking site for cytosolic components that translocate to the plasma membrane, p47phox is the subunit that is responsible for transporting the whole cytosolic complex to the docking site during the activation of the NADPH oxidase. Thus, p47phox is regarded as the organizer subunit because it coordinates the interactions of different NADPH oxidase subunits and allows for the formation of an active complex.

OA is a systemic inflammatory disorder that most commonly targets the joints. The synovial fluid in patients with OA contains large numbers of neutrophils and macrophages, which suggests that these factors may contribute to tissue injury. NADPH oxidase activity and the phosphorylation of p47phox are markedly increased in neutrophils from patients with OA. This upregulation could be due to the actions of pro-inflammatory cytokines, such as TNF-α, which is found in high concentrations in the synovial fluids of these patients [45].

Our study presented, for the first time, that p47phox siRNA-encapsulated PLGA NPs could have therapeutic effects on OA patients, leading to reduced chondrocytic cell death and cartilage damages. Additionally, the administration of NPs to the knees of patients with OA may influence their in vivo efficacy. However, these considerations are beyond the scope of this study and will be investigated in future work from our research group.

## 5. Conclusions

In the present study, the successful encapsulation of siRNA into PLGA NPs resulted in near-monodispersed and spherical particles with a low polydispersity index, and these particles maintained their effects for an extended duration. Furthermore, the present study demonstrated that the p47phox siRNA-encapsulated PLGA NPs acted synergistically to reduce ROS production in an animal model of OA; the formulated PLGA NPs provided a sustained release of siRNA. Additionally, the capability of the manufactured NPs to permit sustained release suggests the potential for a delivery system that could reduce dosing frequency to a weekly regimen. Therefore, p47phox siRNA PLGA NPs may represent a promising novel therapeutic avenue for the treatment of OA.

## Supplementary Materials

The following are available online at https://www.mdpi.com/2073-4360/12/2/443/s1, Figure S1: Intra-articular injection of siRNA p47phox-encapsulated PLGA nanoparticles into the cartilage alleviates mechanical allodynia following MIA-induced OA pain in a dose-dependent manner.

## References

[1] Hoshiyama Y., Otsuki S., Oda S., Kurokawa Y., Nakajima M., Jotoku T., Tamura R., Okamoto Y., Lotz M.K., Neo M. (2015). Chondrocyte clusters adjacent to sites of cartilage degeneration have characteristics of progenitor cells. J. Orthop. Res..

[2] Lee C.M., Kisiday J.D., McIlwraith C.W., Grodzinsky A.J., Frisbie D.D. (2013). Synoviocytes protect cartilage from the effects of injury in vitro. BMC Musculoskelet. Disord..

[3] Sandell L.J., Aigner T. (2001). Articular cartilage and changes in arthritis. An introduction: Cell biology of osteoarthritis. Arthritis Res..

[4] Khan I.M., Palmer E.A., Archer C.W. (2010). Fibroblast growth factor-2 induced chondrocyte cluster formation in experimentally wounded articular cartilage is blocked by soluble Jagged-1. Osteoarthr. Cartil..

[5] Soto-Hermida A., Fernandez-Moreno M., Pertega-Diaz S., Oreiro N., Fernandez-Lopez C., Blanco F.J., Rego-Perez I. (2015). Mitochondrial DNA haplogroups modulate the radiographic progression of Spanish patients with osteoarthritis. Rheumatol. Int..

[6] Henrotin Y., Kurz B., Aigner T. (2005). Oxygen and reactive oxygen species in cartilage degradation: Friends or foes?. Osteoarthr. Cartil..

[7] Lepetsos P., Papavassiliou A.G. (2016). ROS/oxidative stress signaling in osteoarthritis. Biochim. Biophys. Acta.

[8] Altindag O., Erel O., Aksoy N., Selek S., Celik H., Karaoglanoglu M. (2007). Increased oxidative stress and its relation with collagen metabolism in knee osteoarthritis. Rheumatol. Int..

[9] Davies C.M., Guilak F., Weinberg J.B., Fermor B. (2008). Reactive nitrogen and oxygen species in interleukin-1-mediated DNA damage associated with osteoarthritis. Osteoarthr. Cartil..

[10] Dupuy C., Ohayon R., Valent A., Noel-Hudson M.S., Deme D., Virion A. (1999). Purification of a novel flavoprotein involved in the thyroid NADPH oxidase. Cloning of the porcine and human cdnas. J. Biol. Chem..

[11] Geiszt M., Leto T.L. (2004). The Nox family of NAD(P)H oxidases: Host defense and beyond. J. Biol. Chem..

[12] Ma M.W., Wang J., Zhang Q., Wang R., Dhandapani K.M., Vadlamudi R.K., Brann D.W. (2017). NADPH oxidase in brain injury and neurodegenerative disorders. Mol. Neurodegener..

[13] Li J.M., Mullen A.M., Yun S., Wientjes F., Brouns G.Y., Thrasher A.J., Shah A.M. (2002). Essential role of the NADPH oxidase subunit p47(phox) in endothelial cell superoxide production in response to phorbol ester and tumor necrosis factor-alpha. Circ. Res..

[14] Chenevier-Gobeaux C., Lemarechal H., Bonnefont-Rousselot D., Poiraudeau S., Ekindjian O.G., Borderie D. (2006). Superoxide production and NADPH oxidase expression in human rheumatoid synovial cells: Regulation by interleukin-1beta and tumour necrosis factor-alpha. Inflamm. Res..

[15] Sung J.Y., Hong J.H., Kang H.S., Choi I., Lim S.D., Lee J.K., Seok J.H., Lee J.H., Hur G.M. (2000). Methotrexate suppresses the interleukin-6 induced generation of reactive oxygen species in the synoviocytes of rheumatoid arthritis. Immunopharmacology.

[16] Chi P.L., Chen Y.W., Hsiao L.D., Chen Y.L., Yang C.M. (2012). Heme oxygenase 1 attenuates interleukin-1beta-induced cytosolic phospholipase A2 expression via a decrease in NADPH oxidase/reactive oxygen species/activator protein 1 activation in rheumatoid arthritis synovial fibroblasts. Arthritis Rheum..

[17] Drevet S., Gavazzi G., Grange L., Dupuy C., Lardy B. (2018). Reactive oxygen species and NADPH oxidase 4 involvement in osteoarthritis. Exp. Gerontol..

[18] Rousset F., Hazane-Puch F., Pinosa C., Nguyen M.V., Grange L., Soldini A., Rubens-Duval B., Dupuy C., Morel F., Lardy B. (2015). IL-1beta mediates MMP secretion and IL-1beta neosynthesis via upregulation of p22(phox) and NOX4 activity in human articular chondrocytes. Osteoarthr. Cartil..

[19] Lepetsos P., Pampanos A., Lallos S., Kanavakis E., Korres D., Papavassiliou A.G., Efstathopoulos N. (2013). Association of NADPH oxidase p22phox gene C242T, A640G and -930A/G polymorphisms with primary knee osteoarthritis in the Greek population. Mol. Biol. Rep..

[20] Grange L., Nguyen M.V., Lardy B., Derouazi M., Campion Y., Trocme C., Paclet M.H., Gaudin P., Morel F. (2006). NAD(P)H oxidase activity of Nox4 in chondrocytes is both inducible and involved in collagenase expression. Antioxid. Redox Signal..

[21] Clavijo-Cornejo D., Martinez-Flores K., Silva-Luna K., Martinez-Nava G.A., Fernandez-Torres J., Zamudio-Cuevas Y., Guadalupe Santamaria-Olmedo M., Granados-Montiel J., Pineda C., Lopez-Reyes A. (2016). The Overexpression of NALP3 Inflammasome in Knee Osteoarthritis Is Associated with Synovial Membrane Prolidase and NADPH Oxidase 2. Oxid. Med. Cell. Longev..

[22] Van Dalen S.C.M., Kruisbergen N.N.L., Walgreen B., Helsen M.M.A., Sloetjes A.W., Cremers N.A.J., Koenders M.I., van de Loo F.A.J., Roth J., Vogl T. (2018). The role of NOX2-derived reactive oxygen species in collagenase-induced osteoarthritis. Osteoarthr. Cartil..

[23] Chenevier-Gobeaux C., Simonneau C., Therond P., Bonnefont-Rousselot D., Poiraudeau S., Ekindjian O.G., Borderie D. (2007). Implication of cytosolic phospholipase A2 (cPLA2) in the regulation of human synoviocyte NADPH oxidase (Nox2) activity. Life Sci..

[24] Yasuhara R., Miyamoto Y., Akaike T., Akuta T., Nakamura M., Takami M., Morimura N., Yasu K., Kamijo R. (2005). Interleukin-1beta induces death in chondrocyte-like ATDC5 cells through mitochondrial dysfunction and energy depletion in a reactive nitrogen and oxygen species-dependent manner. Biochem. J..

[25] Funato S., Yasuhara R., Yoshimura K., Miyamoto Y., Kaneko K., Suzawa T., Chikazu D., Mishima K., Baba K., Kamijo R. (2017). Extracellular matrix loss in chondrocytes after exposure to interleukin-1beta in NADPH oxidase-dependent manner. Cell Tissue Res..

[26] Yoshimura K., Miyamoto Y., Yasuhara R., Maruyama T., Akiyama T., Yamada A., Takami M., Suzawa T., Tsunawaki S., Tachikawa T. (2011). Monocarboxylate transporter-1 is required for cell death in mouse chondrocytic ATDC5 cells exposed to interleukin-1beta via late phase activation of nuclear factor kappaB and expression of phagocyte-type NADPH oxidase. J. Biol. Chem..

[27] Panday A., Sahoo M.K., Osorio D., Batra S. (2015). NADPH oxidases: An overview from structure to innate immunity-associated pathologies. Cell. Mol. Immunol..

[28] Morozov I., Lotan O., Joseph G., Gorzalczany Y., Pick E. (1998). Mapping of functional domains in p47(phox) involved in the activation of NADPH oxidase by “peptide walking”. J. Biol. Chem..

[29] Kou L., Xiao S., Sun R., Bao S., Yao Q., Chen R. (2019). Biomaterial-engineered intra-articular drug delivery systems for osteoarthritis therapy. Drug Deliv..

[30] Operti M.C., Dolen Y., Keulen J., van Dinther E.A.W., Figdor C.G., Tagit O. (2019). Microfluidics-Assisted Size Tuning and Biological Evaluation of PLGA Particles. Pharmaceutics.

[31] Bakker B., Eijkel G.B., Heeren R.M., Karperien M., Post J.N., Cillero-Pastor B. (2016). Oxygen Regulates Lipid Profiles in Human Primary Chondrocyte Cultures. Osteoarthr. Cartil..

[32] Roh D.H., Kim H.W., Yoon S.Y., Seo H.S., Kwon Y.B., Kim K.W., Han H.J., Beitz A.J., Na H.S., Lee J.H. (2008). Intrathecal injection of the sigma(1) receptor antagonist BD1047 blocks both mechanical allodynia and increases in spinal NR1 expression during the induction phase of rodent neuropathic pain. Anesthesiology.

[33] Shin J., Yin Y., Kim D.K., Lee S.Y., Lee W., Kang J.W., Kim D.W., Hong J. (2019). Foxp3 plasmid-encapsulated PLGA nanoparticles attenuate pain behavior in rats with spinal nerve ligation. Nanomedicine.

[34] Shin N., Kim H.G., Shin H.J., Kim S., Kwon H.H., Baek H., Yi M.H., Zhang E., Kim J.J., Hong J. (2019). Uncoupled Endothelial Nitric Oxide Synthase Enhances p-Tau in Chronic Traumatic Encephalopathy Mouse Model. Antioxid. Redox Signal..

[35] Shin J., Yin Y., Park H., Park S., Triantafillu U.L., Kim Y., Kim S.R., Lee S.Y., Kim D.K., Hong J. (2018). p38 siRNA-encapsulated PLGA nanoparticles alleviate neuropathic pain behavior in rats by inhibiting microglia activation. Nanomedicine.

[36] Peltonen L., Aitta J., Hyvonen S., Karjalainen M., Hirvonen J. (2004). Improved entrapment efficiency of hydrophilic drug substance during nanoprecipitation of poly(l)lactide nanoparticles. AAPS PharmSciTech.

[37] Karim A., Amin A.K., Hall A.C. (2018). The clustering and morphology of chondrocytes in normal and mildly degenerate human femoral head cartilage studied by confocal laser scanning microscopy. J. Anat..

[38] Turrens J.F. (2003). Mitochondrial formation of reactive oxygen species. J. Physiol..

[39] Pitcher T., Sousa-Valente J., Malcangio M. (2016). The Monoiodoacetate Model of Osteoarthritis Pain in the Mouse. J. Vis. Exp..

[40] Jain A., Kunduru K.R., Basu A., Mizrahi B., Domb A.J., Khan W. (2016). Injectable formulations of poly(lactic acid) and its copolymers in clinical use. Adv. Drug Deliv. Rev..

[41] Lababidi N., Sigal V., Koenneke A., Schwarzkopf K., Manz A., Schneider M. (2019). Microfluidics as tool to prepare size-tunable PLGA nanoparticles with high curcumin encapsulation for efficient mucus penetration. Beilstein J. Nanotechnol..

[42] Hunter D.J., Zhang Y., Niu J., Goggins J., Amin S., LaValley M.P., Guermazi A., Genant H., Gale D., Felson D.T. (2006). Increase in bone marrow lesions associated with cartilage loss: A longitudinal magnetic resonance imaging study of knee osteoarthritis. Arthritis Rheum..

[43] Marchi S., Giorgi C., Suski J.M., Agnoletto C., Bononi A., Bonora M., De Marchi E., Missiroli S., Patergnani S., Poletti F. (2012). Mitochondria-ros crosstalk in the control of cell death and aging. J. Signal Transduct..

[44] Zhang X., Yang Y., Li X., Zhang H., Gang Y., Bai L. (2019). Alterations of autophagy in knee cartilage by treatment with treadmill exercise in a rat osteoarthritis model. Int. J. Mol. Med..

[45] El-Benna J., Dang P.M., Gougerot-Pocidalo M.A., Marie J.C., Braut-Boucher F. (2009). p47phox, the phagocyte NADPH oxidase/NOX2 organizer: Structure, phosphorylation and implication in diseases. Exp. Mol. Med..

[46] Faust L.R., El Benna J., Babior B.M., Chanock S.J. (1995). The phosphorylation targets of p47phox, a subunit of the respiratory burst oxidase. Functions of the individual target serines as evaluated by site-directed mutagenesis. J. Clin. Investig..

