# Supplementary material for: p47phox siRNA-Loaded PLGA Nanoparticles Suppress ROS/Oxidative Stress-Induced Chondrocyte Damage in Osteoarthritis
Source: Polymers (Basel). 2020 Feb 13;12(2):443. doi: 10.3390/polym12020443 (PMC7077645; doi:10.3390/polym12020443)
Supplement: Supplementary file 1 [file polymers-12-00443-s001.pdf]

Supplymentray figure 1.

A

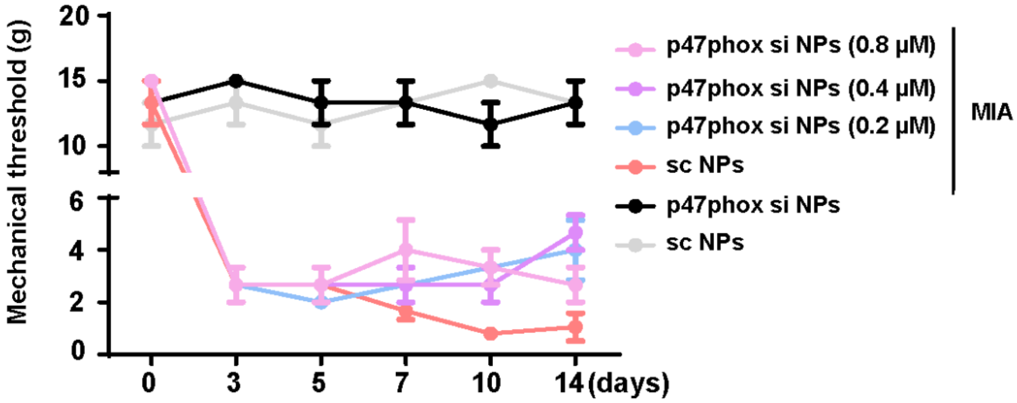

Supplementary Figure 1. Intra-articular injection of siRNA p47phox-encapsulated PLGA nanoparticles into the cartilage alleviates mechanical allodynia following MIA-induced OA pain in a dose-dependent manner. (A) siRNA p47phox NPs with different doses or scrambled siRNA NPs were intra-articular injected into the cartilage of the rats and the von Frey test was conducted to each group of the rats on 3, 5, 7, 10, and 14 days after injection. Data are presented as the mean ±SEM.
